# The Protective Role of Troxerutin (Trox) in Counteracting Anaplastic Thyroid Carcinoma (ATC) Progression

**DOI:** 10.3390/biomedicines12081755

**Published:** 2024-08-05

**Authors:** Valentina Bova, Rossella Basilotta, Giovanna Casili, Marika Lanza, Alessia Filippone, Michela Campolo, Anna Paola Capra, Giulia Vitale, Giulia Chisari, Cristina Colarossi, Dario Giuffrida, Irene Paterniti, Emanuela Esposito

**Affiliations:** 1 Department of Chemical, Biological, Pharmaceutical and Environmental Sciences, University of Messina, Viale F. Stagno D’Alcontres 31, 98166 Messina, Italy; valentina.bova@unime.it (V.B.); rossella.basilotta@unime.it (R.B.); giovanna.casili@unime.it (G.C.); marika.lanza@unime.it (M.L.); alessia.filippone@unime.it (A.F.); mcampolo@unime.it (M.C.); annapaola.capra@unime.it (A.P.C.); giulia.vitale@studenti.unime.it (G.V.); ipaterniti@unime.it (I.P.); 2 Istituto Oncologico del Mediterraneo, Via Penninazzo 7, 95029 Viagrande, Italy; giulia.chisari@grupposamed.com (G.C.); cristina.colarossi@grupposamed.com (C.C.); dario.giuffrida@grupposamed.com (D.G.)

**Keywords:** anaplastic thyroid carcinoma (ATC), troxerutin, antitumoral activity, anti-oxidant activity, chemotherapy

## Abstract

Anaplastic thyroid carcinoma (ATC) is a rare thyroid neoplasm characterized by aggressiveness and a high mortality rate. Troxerutin (Trox) is a bioflavonoid widely found in various fruits and vegetables with numerous protective effects, including anticancer activities. To evaluate the anti-oxidant and anti-inflammatory effect of Trox, in vitro and in vivo studies were conducted in a model of ATC. Human ATC 8305C cell lines were treated with increasing concentrations of Trox (10 μg/mL, 30 μg/mL, 100 μg/mL, 300 μg/mL), and our results revealed that Trox treatment was able to reduce the viability of ATC cells and migratory capacity, reducing the expression of anti-apoptotic factors, such as B-cell lymphoma (bcl-2), and increasing the expression of pro-apoptotic factors, such as Caspase-3 and BID, activating oxidative stress mediators, such as manganese superoxide dismutase (MnSOD), heme oxygenase-1 (HO-1), glutathione (GSH) and reactive oxygen species modulator 1 (ROMO-1). Furthermore, Trox modulates NF-κB pathway markers, such as NIK and TRAF-6. Further confirmation was obtained through in vivo studies, in which Trox treatment, at doses of 12.5, 25 and 50 mg/kg, reduced morphological alteration, decreasing mast cell accumulation. Therefore, the use of Trox could be considered a promising strategy to counteract the progression of ATC.

## 1. Introduction

Thyroid cancer is a common type of cancer worldwide, most often diagnosed in young adults, affecting women more than men [1]. Several risk factors contribute to the onset of this pathology—age, childhood radiation exposure, genetic conditions and many more—in particular, gender, since the female population is more prone to it than the male population [2]. Thyroid cancer is divided into differentiated and undifferentiated; the papillary form and the follicular form belong to the former, which have a high frequency of incidence, while the medullary form and the anaplastic form, with less frequency, belong to the undifferentiated form. Although the incidence of developing thyroid cancer has increased in the last 30 years, the survival rate remains high for the well-differentiated forms (over 90%), while it decreases with the undifferentiated forms, such as medullary and anaplastic [3,4]. There have been many advances in the diagnosis and treatment of thyroid cancer recently, although the main option is a combination of treatments, chosen based on several factors affecting the particular form of thyroid cancer. Among these, the most common treatments include surgical resection, which in turn can be partial or complete, hormonal treatment (thyroxine), use of radioactive iodine, chemotherapy and finally target therapy, which consists of the use of drugs, such as sorafenib, lenvatinib, larotrectinib and vandetanib, which target genes for particular proteins involved in cancer cell survival [5,6]. New anticancer molecules are urgently needed for aggressive, metastatic and advanced thyroid cancers. In recent decades, attention has been paid to phytochemicals, which can guarantee the necessary biochemical variability for therapeutic purposes [7]. There are several bioactive molecules used for the treatment of thyroid cancer, including curcumin, artichoke extract, chrysin and many others, which, thanks to their anti-oxidant, anti-proliferative properties, contribute to slowing down/countering tumor progression [8,9]. In addition to these compounds, another bioactive molecule is Troxerutin (Trox) [10], which is also called Vitamin P4; it is a biflavonoid hydroxyethylrutoside extracted from *Styphnolobium japonicum* [11]. It is present in foods such as tea, coffee, cereals, as well as in a variety of fruits and vegetables [12]. Trox, thanks to its vasoprotective properties, is mainly used in the medical field in all pathologies presenting capillary fragility. Furthermore, it has been demonstrated that it has pleiotropic capacities, i.e., it acts simultaneously on multiple cellular metabolic systems and pathways, exerting anti-inflammatory, vasoprotective, neuroprotective, anti-oxidant and immunomodulatory actions [13]. The anti-proliferative properties of Trox have been investigated in oncology, particularly in neck cancer, liver cancer and non-small-cell lung tumor [14,15]. Therefore, this article aimed to demonstrate the capacity of Trox treatment to exert protective effects in counteracting anaplastic thyroid cancer (ATC).

## 2. Materials and Methods

### 2.1. In Vitro Studies

#### 2.1.1. Materials

Troxerutin was purchased from Sigma-Aldrich (Cat. # PHR2815; St. Louis, MI, USA). Troxerutin was dissolved in water/culture medium. All chemicals were of the highest commercial grade available. All stock solutions were dissolved in nonpyrogenic saline (0.9% NaCl; Baxter Healthcare Ltd., Thetford, Norfolk, UK) or 10% ethanol (Sigma-Aldrich; St. Louis, MI, USA).

#### 2.1.2. Cell Cultures

Human thyroid carcinoma follicular, human anaplastic thyroid carcinoma and human primary papillary thyroid carcinoma FTC-133, 8305C and K1 cell lines, respectively, were obtained from American Type Culture Collection (ATCC, Rockville, MD, USA). FTC-133, 8305C and K1 cells were cultured in RPMI-1640 (Life Technologies, Gibco^®^; Carlsbad, CA, USA) supplemented with Fetal Bovine Serum (FBS, Life Technologies, Gibco^®^; Carlsbad, CA, USA) at 10%, 100 U/mL of penicillin and 100 μg/mL of Streptomycin. The cell lines were maintained in incubators at 37 °C with CO_2_ at 5%.

#### 2.1.3. Experimental Groups

- Control group: FTC-133, 8305C and K1 cell lines were treated with only basal medium RPMI-1640 for 24 h;
- Trox 10 μg/mL: FTC-133, 8305C and K1 cells were treated with Trox 10 μg/mL for 24 h;
- Trox 30 μg/mL: FTC-133, 8305C and K1 cells were treated with Trox 30 μg/mL for 24 h;
- Trox 100 μg/mL: FTC-133, 8305C and K1 cells were treated with Trox 100 μg/mL for 24 h;
- Trox 300 μg/mL: FTC-133, 8305C and K1 cells were treated with Trox 300 μg/mL for 24 h.

For other analyses, we continued to analyze only Trox 100 μg/mL and 300 μg/mL because they represented the most cytotoxic concentrations revealed by the MTT assay. Moreover, since Trox exerted a similar effect on cell viability in between cell lines, we decided to continue the study using only the 8305C cell line.

#### 2.1.4. Cell Viability (MTT Assay)

Cell viability of the FTC-133, 8305C and K1 cells was evaluated using a mitochondria-dependent dye for live cells (Tetrazolium dye; 3-(4,5-dimethylthiazol-2l)-2,5-diphenyltetrazolium bromide assay (MTT assay); M5655; Sigma-Aldrich) [16]. 8305C cells were plated on 96-well plates at a density of 4 × 10^4^ cells/well to a final volume of 150 μL. After 24 h, FTC-133, 8305C and K1 cells were treated with Trox for 24 h at increasing concentrations of 10, 30, 100 and 300 μg/mL, dissolved in basal medium. After 24 h, cells were incubated at 37 °C with MTT (0.2 mg/mL) for 1 h; the medium was removed by aspiration, and then, cells were lysed with DMSO (100 mL). The extent of reduction in MTT to formazan was quantified by measurement of optical density at 540 nm (OD540) with a microplate reader, as previously described.

#### 2.1.5. Wound Healing Assay (Scratch Test)

The scratch assay was performed to study the effects of Trox on 8305C cell migration according to [17]. Briefly, 8305C cells with around 80% confluency were detached, and 2 × 10^6^ 8305C cells were plated on 60 mm plates (Corning Cell Culture, Tewksbury, MA, USA) in a volume of 2 mL. After 24 h, the cell layer was scratched with a p200 pipette tip to create a straight line. Subsequently, debris was removed from each plate and replaced with a normal culture medium in the control group and treated for 24 h with Trox at concentrations of 100 and 300 μg/mL. Finally, each plate was placed under a microscope and photographed. Images of the cells were captured, and the wound width at the marked wound location after 24 h was used to measure the migratory ability of the cells. The cell migration rate was analyzed and calculated using ImageJ (1.53a version) software.

#### 2.1.6. Colony Formation Assay

To determine the tumor response to Trox treatment, colony formation was performed [18]. Human anaplastic thyroid carcinoma 8305C cells were plated into six-well plates at a density of 10^3^ cells/well, in a final volume of 2 mL. Twenty-four hours after the attachment of cells to the dishes, 8305C cells were treated with Trox at concentrations of 100 and 300 μg/mL, dissolved in basal medium and placed in the incubator until the formation of sufficiently large colonies; the control group was treated with solvent alone. After 24 h of treatment, the medium above the cells was removed, and the wells were rinsed carefully with PBS; subsequently, the basal medium was added to FBS 10% of the total volume and incubated for 10 days. After this incubation time, the cells were washed with PBS and stained with 0.1% (*w*/*v*) crystal violet. Finally, the stained cells were imaged using a bright-field microscope [19].

#### 2.1.7. Western Blot Analysis

Western blot analysis using 8305C cell lysates was performed as previously described [20]. 8305C cells were washed twice with ice-cold phosphate-buffered saline (PBS), collected and resuspended in lysis buffer containing 20 mM Tris-HCl, pH 7.4, 10 mM NaF, 150 μL of NaCl, 1% Nonidet P-40 and a protease cocktail of inhibitors (Cat. No. 11836153001; Roche, Basel, Switzerland). After 40 min, cell lysates were centrifuged at 12,000 rpm for 15 min at 4 °C. The protein concentration was estimated using the Bio-Rad protein assay (Bio-Rad Laboratories, Hercules, CA, USA) using bovine serum albumin as a standard. The samples were then heated to 95 °C for 5 min, and equal amounts of proteins were separated via 10–15% sodium dodecyl sulfate-polyacrylamide gel electrophoresis (SDS-PAGE) and transferred to a membrane of polyvinylidene difluoride (PVDF) (Immobilon-P; Cat #88018; Thermofisher Scientific; Waltham, MA, USA). The following primary antibodies were used: anti-nuclear factor of kappa light chain-enhancer in B cells (NF-κB) (1:500; Santa Cruz Biotechnology, Dallas, TX, USA; sc-8008); anti-inhibitor nuclear factor of kappa light chain-enhancer in B cells alpha (IκB-α) (1:500; Santa Cruz Biotechnology, Dallas, TX, USA; sc-1643); anti-bcl2 (1:500; sc-7382; Santa Cruz Biotechnology), anti-BID (1:500; sc-373939; Santa Cruz Biotechnology), anti-Caspase3 (1:500; sc-56053; Santa Cruz Biotechnology) for the apoptotic pathway; anti-NIK (1:500; sc-8417; Santa Cruz Biotechnology), anti-TRAF6 (1:500; sc-8409; Santa Cruz Biotechnology) for the inflammatory pathway; anti-MnSOD (1:500; sc-133134; Santa Cruz Biotechnology), anti-HO1 (1:500; #ALX-210-116; Enzo; Farmingdale, NY, USA) for oxidative stress.

#### 2.1.8. Enzyme-Linked Immunosorbent Assay (ELISA) for IL-12p70, IL-17A, GSH and ROMO-1

The ELISA kit was performed to evaluate the concentration of IL-12p70 (Cat# M1270; Biotechne; Minneapolis, MN, USA) and IL-17A (Cat# KE10020; Proteintech; Rosemont, IL, USA) for the inflammatory response. GSH (ab239327; Abcam; Cambridge, UK) and ROMO-1 (Cat# orb441988; Biorbyt; Cambridge, UK) ELISA kits were performed to study the anti-oxidative response.

#### 2.1.9. Statistical Analysis

All values are expressed as mean ± standard error of the mean (SEM) of “n” observation. Each analysis was performed three times with three sample replicates for each one. The results were analyzed using one-way analysis of variance (ANOVA), followed by a Bonferroni post hoc test for multiple comparisons. A *p*-value of less than 0.05 was considered significant.

### 2.2. In Vivo Studies

#### 2.2.1. Cell Line

The 8305C cell line was cultured in a 75 cm^2^ flask with RPMI-1640 (Life Technologies, Gibco^®^; Carlsbad, CA, USA) supplemented with antibiotics (Penicillin 1000 units-Streptomycin 0.1 mg/L, Sigma-Aldrich, Cat#P4333), L-Glutamine and 10% FBS, in a humidified atmosphere containing 5% CO_2_ at 37 °C.

#### 2.2.2. Animals

BALB/c nude male mice were obtained from Jackson Laboratory (Bar Harbor; Hancock, ME, USA) and housed in microisolator cages under pathogen-free conditions on a 12 h light and 12 h dark schedule for a week. Animals were fed a standard diet and water ad libitum. Animal experiments followed the Italian regulations on the protection of animals used for experimental and other scientific purposes (DM 116192), as well as EU regulations (European Directive 2010/63/EU amended by Regulation 2019/1010), as well as the ARRIVE guidelines. 

#### 2.2.3. Orthotopic Model of ATC

An orthotopic tumor model was performed as previously described [21]. Mice were inoculated with 5 × 10^5^ human 8305C in 0.1 mL of phosphate-buffered saline (PBS) into the right lobe of the thyroid for 13 days. From day 13, mice received oral treatment with Trox daily at doses of 12.5, 25 and 50 mg/kg for 14 days. Finally, on day 28, the animals were sacrificed, and the thyroid gland was surgically removed and analyzed.

#### 2.2.4. Experimental Groups

Mice were subdivided into the following groups:

- Sham group + Veh: Mice received oral administration of the saline solution;
- ATC group + Veh: Mice received tumor cell inoculation;
- ATC + Trox 12.5 mg/kg: Mice were inoculated with tumor cells and received oral administration of Trox 12.5 mg/kg for 14 days;
- ATC + Trox 25 mg/kg: Mice were inoculated with tumor cells and received oral administration of Trox 25 mg/kg for 14 days;
- ATC + Trox 50 mg/kg: Mice were inoculated with tumor cells and received oral administration of Trox 50 mg/kg for 14 days.

#### 2.2.5. Histological Evaluation

Following the secretion, a histological evaluation of the thyroid tissue was performed, as previously described. Briefly, the samples were fixed in 10% neutral formalin for 24 h; subsequently, they were processed and embedded in paraffin and finally sectioned at 7 μm. After deparaffinization, the sections were stained with Hematossilin/Eosin (H&E) [22]. The slides were analyzed by a pathologist blinded to the treatment groups. All sections were analyzed using Nikon Eclipse Ci-L microscope (Nikon Europe B.V; Stroombaan, The Netherland). The images were shown at magnifications of 2× (400 μm of the bar scale) and 20× (50 μm of the bar scale).

#### 2.2.6. Toluidine Blue Staining

Thyroid tissue sections were deparaffinized in xylene and dehydrated by graded successions of ethanol, 5 min in each solution. The sections were then sited in water for 5 min, relocated to toluidine blue for 4 min and then blotted cautiously. Sections were positioned in absolute alcohol for 1 min, cleared in xylene and fixed on glass slides using Eukitt (Bio-Optica, Milan, Italy). The number of metachromatic stained mast cells was obtained by counting five high-power fields (40×) per section using Nikon Eclipse Ci-L microscope [23].

#### 2.2.7. Western Blot Analysis of NF-κB/IκB-α Pathway, IL-12A p35, IL-6, IL-17A, Caspase3, p53, BID and bcl-2 in Thyroid Tissue

The tumor samples from each mouse were suspended in extraction Buffer A (0.2 mM PMSF, 0.15 mM pepstatin A, 20 mM leupeptin, 1 mM sodium orthovanadate), homogenized at the highest setting for 2 min and centrifuged at 12,000 rpm for 4 min at 4 °C, as previously described [20]. The supernatants were the cytosolic fraction, whereas the pellets, containing enriched nuclei, were resuspended in Buffer B (1% Triton X-100, 150 mM NaCl, 10 mM Tris–HCl, pH 7.4, 1 mM EGTA, 1 mM EDTA, 0.2 mM PMSF, 20 mm leupeptin, 0.2 mM sodium orthovanadate) and centrifuged at 12,000 rpm for 10 min at 4 °C; the supernatants were the nuclear fraction. Protein concentration was estimated by the Bio-Rad protein assay using bovine serum albumin as a standard. Then, tumor samples, in equal amounts of protein, were separated on 12% SDS-PAGE gel and transferred to a nitrocellulose membrane, as previously described. The following primary antibodies were used: anti-NF-κB (1:500; Santa Cruz Biotechnology, Dallas, TX, USA; sc-8008) for the nuclear fraction; anti-IκBα (1:500; Santa Cruz Biotechnology, Dallas, TX, USA; sc-1643); anti-IL12A p35 (1:500; Santa Cruz Biotechnology, Dallas, TX, USA; sc-517382); anti-IL6 (1:500; Santa Cruz biotechnology; Dallas, TX, USA; sc-57315); anti-IL17A (1:500; Santa Cruz Biotechnology; Dallas, TX, USA; sc-374218); anti-caspase3 (1:500; Santa Cruz biotechnology; Dallas, TX, USA; sc-56053); anti-p53 (1:500; Santa Cruz Biotechnology; Dallas, TX, USA; sc-126); anti-BID (1:500; Santa Cruz Biotechnology, Dallas, TX, USA; sc-373939); anti-bcl2 (1:500; Santa Cruz Biotechnology, Dallas, TX, USA; sc-7382) for the cytosolic fraction. Antibody dilutions were performed in PBS/5% *w*/*v* non-fat dried milk/0.1% Tween-20 (PMT), and membranes were incubated overnight at 4 °C. Membranes were then incubated with a secondary antibody (1:2000, Jackson ImmunoResearch, West Grove, PA, USA) for 1 h at room temperature. To ascertain that these blots were loaded with equal amounts of protein lysate, they were also incubated with β-actin antibody (for the cytosolic fraction 1:500; Santa Cruz Biotechnology, Dallas, TX, USA; sc-8432) or lamin A/C (for the nuclear fraction 1:500, Santa Cruz Biotechnology, Dallas, TX, USA; sc-376248). Signals were detected with an enhanced chemiluminescence (ECL) detection system reagent, according to the manufacturer’s instructions (Thermo Fisher, Waltham, MA, USA). The relative expression of the protein bands was quantified via densitometry with BIORAD ChemiDocTMXRS + software 6.1. The densitometric values were normalized with lamin A/C and β-actin and expressed as % of the control.

#### 2.2.8. Immunolocalization of Ki67 in Thyroid Tissue

Immunohistochemical localization was performed, as previously described [24]. Slides were incubated overnight (O/N) using the following primary antibodies: anti-Ki67 (Cat #MA5-14520; 1:100 in PBS, *v*/*v*; Invitrogen; Waltham, MA, USA). At the end of incubation with the primary antibodies, the sections were washed with PBS and incubated with a secondary antibody (Santa Cruz Biotechnology, Dallas, TX, USA) for 1 h at room temperature. The reaction was revealed via a chromogenic substrate (brown DAB), and counterstaining with nuclear fast-red was performed. Immunohistochemical images were obtained and observed using a Nikon Eclipse Ci-L microscope. The percentage area of immunoreactivity (brown staining, determined by the number of positive cells) was expressed as % of the total tissue area (red staining) of five random fields with an objective lens at 20× magnification, and the analysis was performed using ImageJ. Densitometry analysis was performed using GraphPad version 8.0 (La Jolla, CA, USA). Images are shown at 20× and 40× magnification.

## 3. Results

### 3.1. Trox Treatment Reduced FTC-133, 8305C and K1 Viability in a Concentration-Dependent Manner

**Figure 1:**
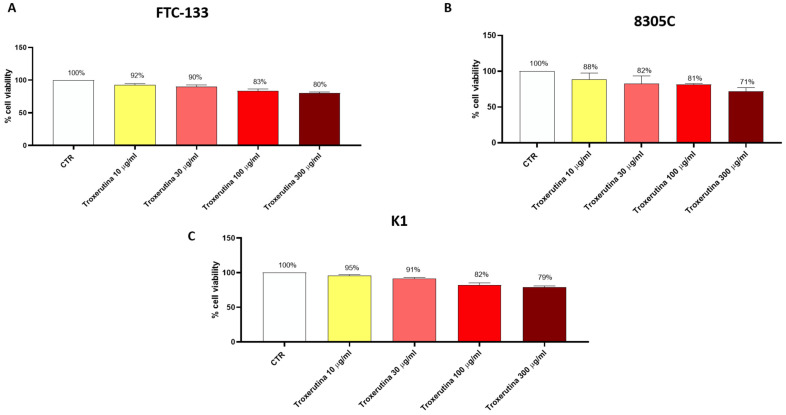
Trox treatment reduced the viability of FTC-133, 8305C and K1 cell lines. MTT assay revealed that the treatment with Trox at the concentration of 10, 30, 100 and 300 μg/mL significantly reduced viability in the FTC-133, 8305C and K1 cell lines in a concentration-dependent manner at 24 h (**A**–**C**) compared to the control group. Data are representative of at least three independent experiments.

FTC-133, 8305C and K1 cells were treated with Trox at concentrations of 10, 30, 100 and 300 μg/mL for 24 h to determine the concentration that caused toxicity in these cells. We observed a decrease in FTC-133, 8305C and K1 viability in a concentration-dependent manner (Figure 1A–C), suggesting that Trox was able to inhibit FTC-133, 8305C and K1 proliferation. Moreover, Trox showed similar effects on cell viability in all thyroid cancer cells; consequently, we decided to study the effect of Trox on the 8305C cell line alone, at concentrations of 100 and 300 μg/mL. The 8305C cell line was chosen because it represents the aggressive form of thyroid cancer, with a minor percentage of frequency (≤1%).

### 3.2. Trox Treatment Inhibited 8305C Cell Migration

**Figure 2:**
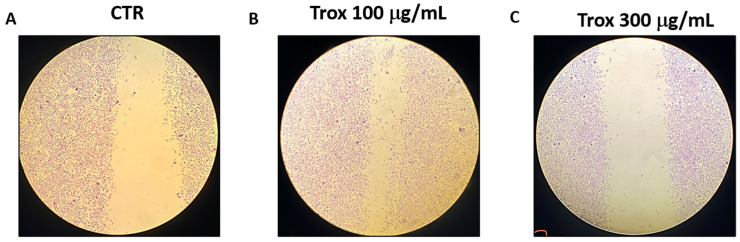
Trox treatment reduced the migration capability of 8305C cells. 8305C cells were examined for cell invasion in 60 mm plates by carrying out the scratch test (**A**–**C**). The wound was photographed 24 h after the Trox treatments to measure the migratory capacity of the cells. The 100 and 300 μg/mL concentrations of Trox significantly affected the cell migration rate, reducing the invasiveness of 8305C cells compared to untreated cells (**B**,**C**).

To evaluate the invasion capacity of 8305C cells, the scratch test was performed. We found that Trox, at the concentrations of 100 and 300 μg/mL, was more able to reduce the migration rate of cells into the scratch area than the control group that received only basal medium (Figure 2).

### 3.3. 8305C Cell Proliferation Decreased after Treatment with Trox

**Figure 3:**
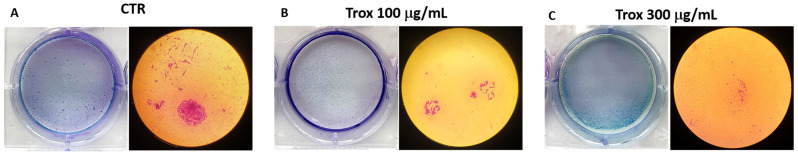
Proliferation properties were reduced following Trox treatment. Proliferation of 8305C cells was examined by a colony formation assay in six-well plates (**A**–**C**). After 10 days, the plate was photographed, and the colonies were counted. Trox at concentrations of 100 and 300 μg/mL reduced the ability of cells to proliferate (**B**,**C**), reducing the number of colony formations compared to the control group (**A**).

8305C cell viability was reduced using high concentrations of Trox. To further confirm the ability of Trox to notably reduce cell migration and proliferation, the colony formation assay was also performed. The results obtained show that, after 10 days of incubation with the basal medium and the addition of a 10% FBS supplement, compared to the control group, the cells treated with Trox at concentrations of 100 and 300 μg/mL showed a lower capacity for colony formation (Figure 3B,C). These data therefore suggest that Trox was able to limit cell migration and proliferation.

### 3.4. Trox Treatment Modulated the Inflammatory Response

**Figure 4:**
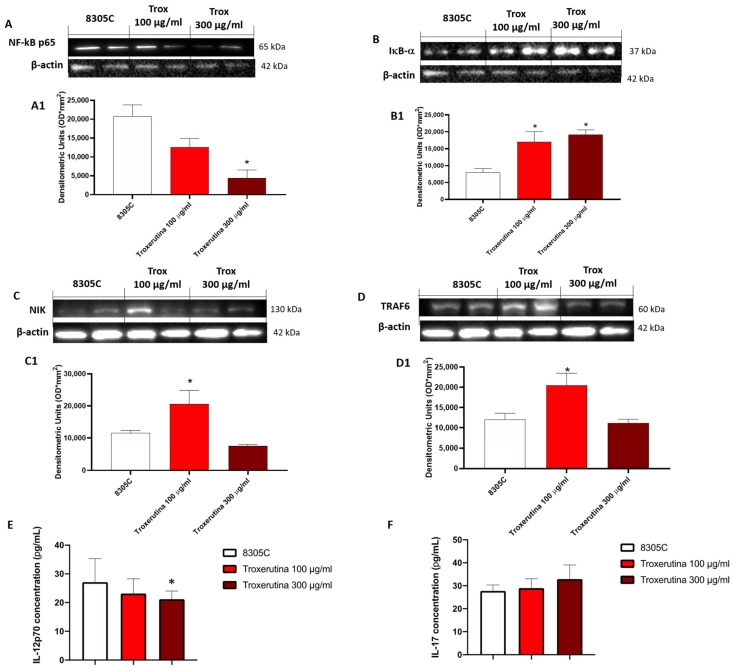
Trox treatment reduced the inflammatory response modulating the NF-κB pathway in 8305C cell lysates. The treatment with Trox at the concentrations of 100 and 300 μg/mL significantly reduced NF-κB/IκB-α pathway (Figure 4). IκB-α expression was increased ((**B**), densitometric analysis (**B1**)), while NF-κB, NIK and TRAF6 expression was reduced ((**A**,**C**,**D**), densitometric analysis (**A1**,**C1**,**D1**)). In the same way, IL12p70 and IL-17 concentration was evaluated by the ELISA kit assay. Our data demonstrated that Trox, at the concentrations of 100 and 300 μg/mL, reduced the concentration of these pro-inflammatory cytokines (**E**,**F**). * *p* < 0.05 vs. 8305C.

The effect of Trox on the NF-κB pathway was evaluated in 8305C cell lysates. Our results demonstrated that the concentration of 100 and 300 μg/mL of Trox was able to reduce NF-κB expression in 8305C (Figure 4A, densitometric analysis A1) but not in 8305C (control group) (* *p* < 0.05 vs. 8305C), which showed a high expression of NF-κB. Contrarily, the Trox treatment restored the IκB-α expression after treatment (Figure 4B, densitometric analysis B1), suggesting that Trox at concentrations of 100 and 300 μg/mL can reduce the inflammatory response. Moreover, the other NIK and TRAF6 expression levels were detected with Western blot analysis (Figure 4C and D, densitometric analysis C1 and D1, respectively). Both NIK and TRAF6 interact directly with some receptors through an intermediate protein, leading to the activation of the immune response [25], such as dendritic cells (DCs) and naïve T cells [25], and supporting the tumor progression [26]. In this analysis, the expression of NIK and TRAF6 was reduced after treatment with Trox (Figure 4C and D, densitometric analysis C1 and D1, respectively) (* *p* < 0.05 vs. 8305C). The ELISA kit assay for IL-12p70 and IL-17 was performed to confirm the anti-inflammatory effect of Trox (* *p* < 0.05 vs. 8305C) (Figure 4E,F).

### 3.5. The Pro-Apoptotic Effects of Trox Treatment

**Figure 5:**
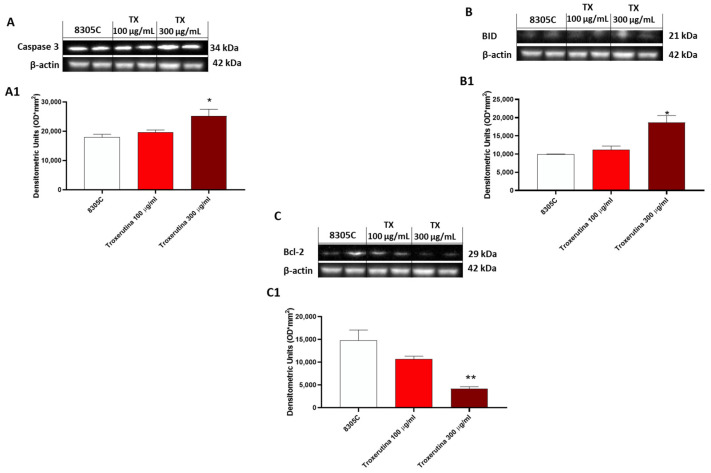
Trox-induced apoptosis processes increased 8305C mortality. Western blot analysis revealed that Trox, at the concentrations of 100 and 300 μg/mL, induced the expression of pro-apoptotic proteins Caspase3 and BID ((**A**,**B**), densitometric analysis (**A1**,**B1**)) compared with the control group. Bcl-2 expression was reduced after 24 h of treatment with Trox ((**C**), densitometric analysis (**C1**)) compared with the control group. * *p* < 0.05 vs. 8305C; ** *p* < 0.01 vs. 8305C.

In 8305C cells, the expression of pro-apoptotic proteins, such as Caspase3 and BID, was found to be low in control tumor cells, while treatment with Trox at the concentrations of 100 and 300 μg/mL showed an increased expression of Caspase3 and BID (* *p* < 0.05 vs. 8305C) (Figure 5A and B, densitometric analysis A1 and B1, respectively). Contrarily, bcl-2 expression was more highly expressed in 8305C cells compared to cells treated with Trox (** *p* < 0.01 vs. 8305C) at the concentrations of 100 and 300 μg/mL (Figure 5C, densitometric analysis C1).

### 3.6. Trox Treatment Modulated Oxidative Stress

**Figure 6:**
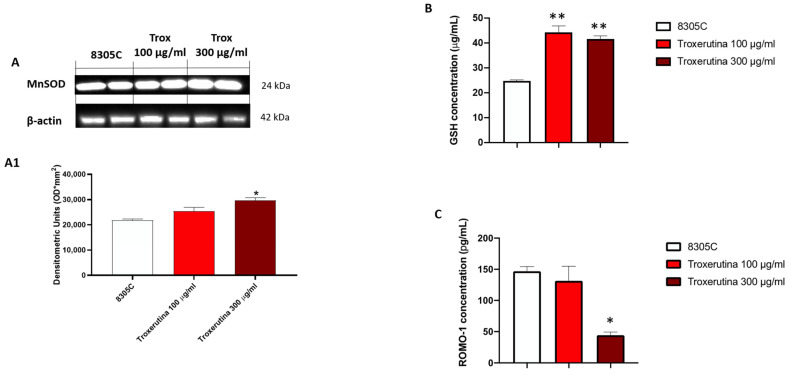
Trox reduced the production of free radicals and favored the expression of anti-oxidative proteins. Our data demonstrated via Western blot analysis of the 8305C cell lysates that Trox at the concentrations of 100 and 300 μg/mL favored the expression of MnSOD ((**A**), densitometric analysis (**A1**)). The ELISA kit assay showed that Trox increased the concentration of anti-oxidant protein GSH (**B**) and reduced the concentration of pro-oxidative protein ROMO1 (**C**). * *p* < 0.05 vs. 8305C; ** *p* < 0.01 vs. 8305C.

To study the anti-oxidative activity of Trox treatment on 8305C cells, we evaluated MnSOD expression via Western blot analyses. MnSOD protein levels appeared to be increased in 8305C cells treated with Trox at concentrations of 100 and 300 μg/mL compared to control tumor cells (Figure 6A) (* *p* < 0.05 vs. 8305C). Finally, to confirm the ability of Trox at concentrations of 100 and 300 μg/mL to modulate oxidative stress, the ELISA kit for GSH and RHOMO-1 was performed. Considering the anti-oxidant properties of GSH, its concentration in cell lysates was evaluated using an ELISA kit. Our results demonstrated that Trox at concentrations of 100 and 300 μg/mL was able to increase the concentration of GSH (** *p* < 0.01 vs. 8305C), further enhancing the anti-oxidant response (Figure 6B). On the contrary, to evaluate the presence of reactive oxygen species, such as the production of free radicals, as a consequence of the marked inflammatory process promoting tumor microenvironment, the concentration of ROMO-1 was estimated using an ELISA kit (Figure 6C). The data obtained demonstrated that Trox, at the higher concentration of 300 μg/mL, was able to better reduce the concentration levels of ROMO-1 compared to untreated 8305C cells (* *p* < 0.05 vs. 8305C) (Figure 6C). Therefore, these results further confirm the ability of Trox to reduce the oxidative response.

### 3.7. Effect of Trox Treatment on Tumor Growth

**Figure 7:**
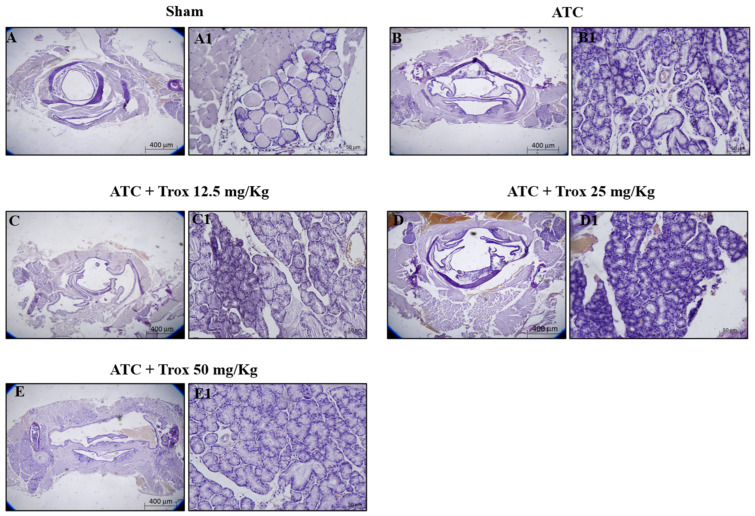
Trox treatment reduced tumoral progression in thyroid tissue. In the orthotopic model, H/E showed that the ATC group presents morphological alteration than the healthy control group (sham) (**A**–**B1**). Trox at doses of 12.5, 25 and 50 mg/kg reduced the characteristics of lymphocytic thyroiditis (**C**–**E1**) compared with the ATC group (**B**,**B1**) in a dose-dependent manner. Moreover, oral treatment with Trox restored the morphological transition in mice after treatment. The results of histological evaluations are displayed at 2× and 20× magnifications.

The histological evaluation showed characteristics of lymphocytic thyroiditis, with the presence of a dense and monotonous mass of cells and morphological transition in mice without treatment (Figure 7B,B1) compared to the healthy control group (Figure 7A,A1). Treatment with Trox at doses of 12.5, 25 and 50 mg/kg was able to reduce this characteristic in a dose-dependent manner, promoting tumor regression (Figure 7C–E1).

### 3.8. Trox Treatment Reduced the Accumulation of Mast Cells in Thyroid Tissue

**Figure 8:**
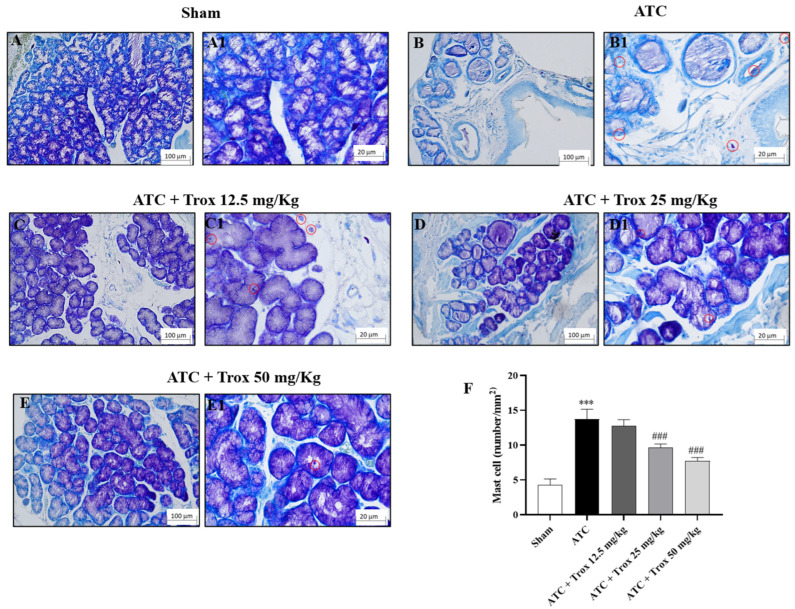
Reduction in the number of mast cells following Trox treatment. Trox treatment at doses of 12.5, 25 and 50 mg/kg reduced the accumulation of mast cells ((**C**–**E1**), score **F**) compared to the ATC group that showed a high number of mast cells ((**B**,**B1**), score **F**). The sham group did not present inflamed tissue and was therefore devoid of mast cells ((**A**,**A1**), score **F**). The results of histological evaluations are displayed at 10× and 40× magnifications. *** *p* < 0.001 vs. sham; ### *p* < 0.001 vs. ATC. Red circle indicated the mast cells localization (**B1**–**E1**).

The thyroid tissue in the presence of a tumor is characterized by an accumulation of infiltrated cells (Figure 8), which therefore contribute to defining the inflammatory state of the tissue. In this regard, toluidine blue staining reported the presence of mast cells in the group of animals with tumor only (*** *p* < 0.001 vs. sham) (Figure 8B,B1, score F). Oral treatment with Trox, only at a dose of 25 and 50 mg/kg, was able to reduce infiltrate accumulation (### *p* < 0.001 vs. ATC) (Figure 8D–E1, score F).

### 3.9. Effect of Trox Treatment on NF-κB/IκB-α Pathway

**Figure 9:**
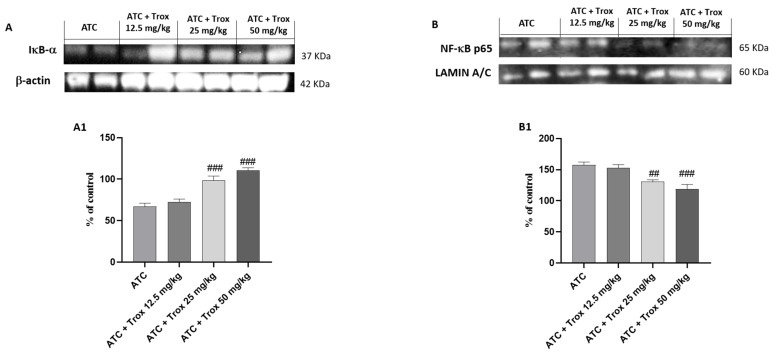
Trox treatment reduced the inflammatory state of the thyroid tissue. To confirm the in vitro studies, Western blot analysis of the thyroid tissue demonstrated that Trox, especially at the doses of 25 and 50 mg/kg, was able to increase the IκB-α levels ((**A**), densitometric analysis (**A1**)) and reduced the expression of NF-κB ((**B**), densitometric analysis (**B1**)) compared with the ATC group ((**A**,**B**), densitometric analysis (**A1**,**B1**)). ## *p* < 0.01 vs. ATC; ### *p* < 0.001 vs. ATC.

Tumors are characterized by NF-κB activation, leading to abnormal proliferation, metastatic spread and resistance to cell death [27]. Based on the data obtained in the in vitro study, the effect of Trox treatment on the 8305C orthotopic model was examined via Western blot analysis on the NF-κB/IκB-α pathway. Therefore, the results obtained demonstrate that oral treatment with Trox for 14 days was able to protect from the cytosolic degradation of IκB-α (### *p* < 0.001 vs. ATC) (Figure 9A, densitometric analysis A1) and prevented the nuclear translocation of NF-κB p65 (## *p* < 0.01 vs. ATC; ### *p* < 0.001 vs. ATC) (Figure 9B, densitometric analysis B1), reducing the inflammatory response of the thyroid tissue.

### 3.10. Trox Treatment Reduced Cytokine Cascade of IL-12A p35, IL-6 and IL-17A

**Figure 10:**
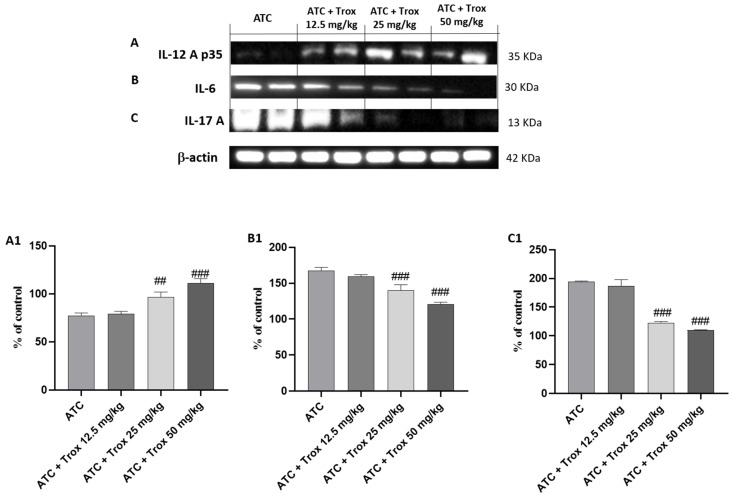
Treatment with Trox reduced the cascade of pro-inflammatory cytokines release. Western blot analysis demonstrated that Trox at doses of 12.5, 25 and 50 mg/kg increased the levels of IL-12Ap35 ((**A**), densitometric analysis (**A1**)) and reduced the expression of IL6 and IL-17A ((**B**,**C**), densitometric analysis (**B1**,**C1**)) compared with the ATC group ((**A**–**C**), densitometric analysis (**A1**–**C1**)). ## *p* < 0.01 vs. ATC; ### *p* < 0.001 vs. ATC.

The activation of NF-κB by the diseased tissue determines the cascade release of pro-inflammatory cytokines, such as IL-6 and IL-17A [28], as can be seen in the ATC group (Figure 10B and C, densitometric analysis B1 and C1, respectively). Regarding the role of IL-12A p35, its expression levels are weakly expressed in undifferentiated thyroid carcinoma (ATC group) (Figure 10A, densitometric analysis A1). Oral treatment with Trox reduced the inflammatory response (## *p* < 0.01 vs. ATC; ### *p* < 0.001 vs. ATC), modulating the cascade of pro-inflammatory cytokines (Figure 10A–C, densitometric analysis A1–C1).

### 3.11. Effect of Trox Treatment on the Apoptotic Process in the 8305C Orthotopic Model

**Figure 11:**
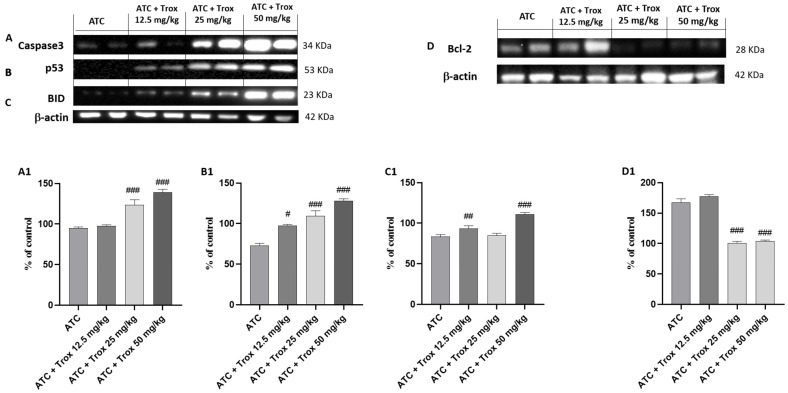
Trox-induced apoptotic process. Our data showed that Trox treatment, especially at high doses of 25 and 50 mg/kg, induced the apoptotic process increasing the expression of Caspase3, p53 and BID ((**A**–**C**), densitometric analysis (**A1**–**C1**)) compared with the ATC group. Moreover, bcl-2 expression was reduced after treatment with Trox ((**D**), densitometric analysis (**D1**)) compared with the ATC group. # *p* < 0.05 vs. ATC; ## *p* < 0.01 vs. ATC; ### *p* < 0.001 vs. ATC.

In tumor progression, both inflammation and apoptosis play an extremely important role. Although in certain pathological conditions, the activation of NF-κB leads to the activation of biochemical and molecular mechanisms favoring cell death by apoptosis, in the tumor context, active NF-κB promotes cell proliferation by reducing apoptosis and increasing the survival of tumor cells [27]. In this regard, the expression levels of pro-apoptotic and anti-apoptotic proteins Caspase3, p53, BID and bcl-2 in the thyroid tissue were studied via Western blot analysis. The results obtained demonstrated a significant increase in three pro-apoptotic proteins Caspase3, p53 and BID in Trox-treated mice (## *p* < 0.01 vs. ATC; ### *p* < 0.001 vs. ATC) (Figure 11A–C, densitometric analysis A1–C1) compared with untreated mice (Figure 11A–C, densitometric analysis A1–C1). In the same manner, oral treatment with Trox, especially at doses of 25 and 50 mg/kg, reduced the expression of bcl-2 (### *p* < 0.001 vs. ATC) (Figure 11D, densitometric analysis D1) compared with the ATC group (Figure 11D, densitometric analysis D1).

### 3.12. Reduction in Proliferative Marker Ki67-Positive Cells following Trox Treatment

**Figure 12:**
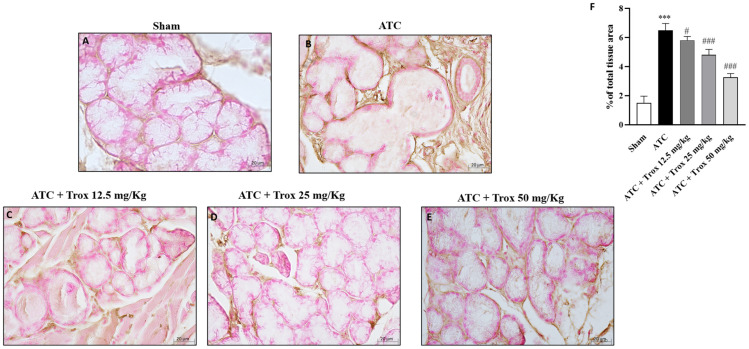
Troxerutin treatment reduced Ki67-positive cells. Ki67 expression was evaluated via histochemistry staining. The ATC group presented a high number of ki67-positive cells ((**B**), score **F**) compared with the control group (**A**), which did not present immunopositivity for the Ki67 marker. Treatment with Trox at doses of 12.5, 25 and 50 mg/kg reduced the number of Ki67-positive cells ((**C**–**E**), score **F**). The results of histological evaluation are displayed at 40× magnifications. *** *p* < 0.001 vs. sham; # *p* < 0.05 vs. ATC; ### *p* < 0.001 vs. ATC.

For decades, the Ki67 protein has been used as a biomarker of cell proliferation in most human and animal cancers, including anaplastic thyroid carcinoma. Moreover, a correlation between Ki67 and the inflammatory state in thyroid tissue has been reported, although the mechanism is still unknown [29]. In this regard, immunohistochemical analysis was performed on thyroid tissue sections for Ki67. The results obtained demonstrated that the group that did not receive the Trox treatment had a high density of Ki67-positive cells (*** *p* < 0.001 vs. sham) (Figure 12B, score F) compared to the healthy control group (Figure 12A, score F). Oral treatment with Trox dose-dependently reduced the immunopositivity of cells for Ki67 (# *p* < 0.05 vs. ATC; ### *p* < 0.001 vs. ATC) (Figure 12C–E, score F).

## 4. Discussion

Anaplastic thyroid cancer, also known as undifferentiated thyroid cancer, is a rare, highly aggressive malignant tumor and represents one of the diseases with the greatest lethal risk and poor prognosis. The Memorial Sloan Kettering Cancer Center (MKKCC) revealed that there are two common subtypes of thyroid cancer associated with transformed ATC subtypes [30]: poor differentiated thyroid cancer (PDTC) and the tall cell variant of papillary DTC [31]. These associations are caused by the mutation of *BRAF* and *RAS* genes, while the mutation in tumor protein p53 (TP53) and telomerase reverse transcriptase (TERT) might help the tumor progression toward the ATC subtype [32]. Furthermore, anaplastic thyroid cancer can spread metastatically to the lymph nodes [33], which is observed in almost 40% of patients [34], and the main regions include the lungs (80%), brain and bones (5–15%). Recently, a screening of natural compounds was performed [35] to identify several novel inhibitors [36]. In this context, we focused on the study of the role of flavonoids, in particular of Trox, in the oncological field, already known for its excellent vasoprotective properties, which have been deepened [37]. Although the activity of Trox in various types of cancer, such as liver cancer, non-small-cell lung cancer, etc., has been evaluated in the past, studies have not yet been conducted on anaplastic thyroid cancer. Initially, the first-time screening of the Trox effect was conducted in three cell lines (FTC-133; 8305C and K1) to obtain a concentration response to select the most cytotoxic concentrations revealed by the MTT assay. These cell lines are the most common in vitro models used to study thyroid cancer [36] because they maintain the tract of human cancer cells, although each of them represents a specific and different type of thyroid cancer.

The data obtained from this first study demonstrated that Trox reduced the viability of FTC-133, 8305C and K1 cells in a concentration-dependent manner at 24 h for the three cell lines, thus enhancing their cytotoxic effect. From the moment that Trox exerted the same effect on all cell lines, and knowing that 8305C cells represent the most aggressive type of thyroid cancer, this study was conducted using only 8305C cell lines.

Furthermore, considering the marked property of the anaplastic thyroid carcinoma cells of proliferation and invasiveness, the cell migration test demonstrated that Trox, at concentrations of 100 and 300 μg/mL, was able to block the invasiveness to adjacent tissues compared with the control group, characterized by untreated 8305C cells, which grew by shortening the gap-scratching distance. Further confirmation was obtained with the colony formation test, which demonstrated the ability of Trox to slow down the proliferative capacity of 8305C cells. It is now known that most cancer cells are characterized by a high inflammatory state [38], and, in this regard, the ability of NF-κB to suppress apoptosis and regulate cell cycle transition was demonstrated, confirming its major role in oncogenesis. In this work, we evaluated the non-canonical kinase (NIK) pathway that induces NF-κB and the expression of the adapter protein TRAF6 widely involved in tumorigenesis. Therefore, the results obtained in this study demonstrated that treatment with Trox at concentrations of 100 and 300 μg/mL was able to modulate the pathway of NF-κB/IκB-α, NIK and TRAF6, reducing the expression of proteins involved in inflammation, favoring instead the upregulation of IκB-α. Furthermore, once activated, NF-κB determined the release of pro-inflammatory cytokines [39], including IL-6 and IL-17A, while IL-12A p35 expression levels were decreased in this study. It was observed that Trox reduced the expression levels of pro-inflammatory cytokines, confirming the ability to reduce the inflammatory state that contributes to tumor progression. Suppression of apoptosis in anaplastic thyroid cancer cells promotes their survival, contributing to pathogenesis. Therefore, based on the studies present in the literature on the ability of Trox to act on the apoptotic process in various pathologies, in this work, we demonstrated that Trox at concentrations of 100 and 300 μg/mL increases the expression of the most involved pro-apoptotic proteins, such as Caspase3 and BID, instead reducing the expression of the anti-apoptotic protein bcl-2, more expressed in the treatment-free 8305C control cells, thus inducing apoptosis in anaplastic thyroid carcinoma cells [40]. Trox is well known for its anti-oxidant properties, and the activation of NF-κB determines the aberration of apoptosis in cancer cells, favoring a condition of oxidative stress in which there is a release of free radicals. In this regard, the expression level of MnSOD was evaluated, which, in some types of tumors, including non-aggressive forms of thyroid cancer, is highly expressed in untreated cells [41], thus favoring tumor growth. While untreated anaplastic thyroid carcinoma cells (8305C) assume a highly aggressive form, their expression is downregulated. Conversely, Trox induced an increase in MnSOD expression levels, thereby reducing the proliferation of 8305C tumor cells and improving the mortality rates [42]. In parallel, the concentration levels of GSH and ROMO1, two important anti- and pro-oxidant markers, were evaluated [43,44]. Our results demonstrated that Trox at concentrations of 100 and 300 μg/mL was able to increase GSH concentration, further enhancing the anti-oxidant response. At the same time, Trox reduced the concentration of ROMO1 compared to untreated 8305C control cells, reducing the production of free radicals, as a consequence of the inflammatory state that favors the tumor microenvironment [45]. Furthermore, to reproduce a valid ATC model, an orthotopic murine model was performed. The 8305C cells created a considerable tumor mass in 20 days from the implant, showing histopathological characteristics of high-grade neoplasia (high proliferative activity and invasiveness), allowing the evaluation of the effect of Trox in counteracting tumor progression, thus confirming the ability of Trox to modulate the activation of the NF-κB/IκB-α pathway. Furthermore, the effect of Trox in counteracting tumor progression was also studied in the in vivo model, thus confirming the ability of Trox to modulate the activation of the NF-κB/IκB-α pathway. Furthermore, active NF-κB can regulate several pathways that promote tumor growth [27]. In particular, once activated, NF-κB determines the activation and release of numerous pro-inflammatory proteins and infiltrated cells, which accumulate in correspondence with the damaged tissue [46,47]. The orthotopic model reported that treatment with Trox reduced the number of mast cells in the thyroid tissue. As described above, active NF-κB results in the cascade release of pro-inflammatory cytokines, such as IL-12A p35, IL-6 and IL-17A [48]. In particular, oral treatment with Trox at doses of 12.5 and 50 mg/kg increased the protein expression of IL-12A p35, reducing the expression of IL-6 and Il-17A, further confirming its ability to modulate the inflammatory response. Furthermore, the activation of NF-κB induces the aberration of apoptosis, which also plays an important role in tumorigenesis. In this work, it was reported that Trox was able to significantly increase the expression levels of pro-apoptotic proteins, such as Caspase3, p53 and BID, decreasing the expression of anti-apoptotic proteins, such as bcl-2 [49]. Numerous studies previously conducted on various tumor forms have highlighted the involvement of the proliferative biomarker Ki67 [50] and its correlation with the inflammatory state indicated by the activation of NF-κB [51]. Thus, the elevated expression of Ki67 in the thyroid tissue in the presence of tumor revealed that treatment with Trox in the orthotopic model was able to reduce its expression, and consequently, tumor growth.

## 5. Conclusions

The in vitro study and the orthotopic mouse model represent the most often performed studies to evaluate the biological mechanisms of tumors and develop probable new compounds (both of natural and synthetic origin) capable of counteracting their progression and being used as adjuvants to the most commonly used therapies. The preliminary data obtained in this first study satisfied the endpoints set by our working group; therefore, our results suggest that it would be worthwhile to study the possibility of using Trox as a treatment modality in ATC in humans. Supported by sources in the literature [36,52], the preclinical study presents a particular limitation due to the probable and involuntary variables found during the experimentation—among these, the genomic instability of the cells themselves due to the passage number used in the study (we used passage number = 31), which is why further studies will be necessary in this regard to evaluate whether the effect of Trox remains unchanged even in other passage numbers.

## Data Availability

The original contributions presented in the study are included in the article, further inquiries can be directed to the corresponding author.
